# Measles and rubella serosusceptibity among population vaccinated with different schedules: the potential impact on measles-rubella elimination in Iran

**DOI:** 10.1186/s12879-021-05970-7

**Published:** 2021-03-25

**Authors:** Hana Saffar, Maryam Khalifeloo, Mohammad-Jafar Saffar, Alireza Abdollahi, Mohammad-Reza Parsaei, Gholam-Reza Ghorbani, Samaneh Salarvand, Mohsen Aarabi

**Affiliations:** 1 Department of Pathology, School of medicine, IKHC, Teheran University of Medical Sciences, Tehran, Iran; 2 Pediatric Infectious Diseases Research Center, communicable Diseases Institute, and Department of Pediatric Infectious Diseases, Bu-Ali Sina Hospital, Mazandaran University of Medical Sciences, Pasdaran Bolv, Sari, Iran; 3 Deputy of Health, Mazandaran University of Medical Sciences, Sari, Iran

**Keywords:** Measles, Rubella, MMR, Measles elimination, Congenial rubella syndrome. Iran

## Abstract

**Background:**

Iranian children were vaccinated with the scheduled two doses of monovalent measles vaccine (mMV) from 1984. In December 2003, a nationwide campaign of measles-rubella (MR) immunization was established to vaccinate 5–25 year- old individuals. In 2004, the mMV was replaced with measles- mumps- rubella (MMR) vaccine. Despite the high vaccination coverage, the outbreaks of measles still occur in the country. In this Study, the MR immunity status of various age groups, vaccinated with different schedules was investigated, and the immunologic response of seronegative subjects to revaccination was examined.

**Methods:**

This cross-sectional study was conducted among 7–33-year-old healthy individuals with a documented history of measles vaccination from November 2017 to June 2018. The subjects were categorized as follows: group A, including 20–33 year-old individuals; vaccinated with 1–2 doses of mMV at ages 9 and 15 months, and revaccinated with MR, group B, including 15–19-year-old individuals, vaccinated with two doses of mMV at 9 and 15 months of age, and received additional dose of MMR upon school entrance, group C, including 11–14 year-old individuals, vaccinated with two-doses of MMR at the ages of 15 months and 6 years, and group D, including 7–10 year-old individuals vaccinated with two-doses of MMR vaccine at the ages 12 and 18 months, respectively. Levels of antimeasles- antirubella IgG antibodies in the collected sera were measured. Also antimeasles- antirubella IgM and IgG of seronegative individuals were reexamined at 4–6 weeks after MMR revaccination. The collected data were analyzed using descriptive statistical methods.

**Results:**

A total of 635 individuals were investigated in this study. Group A, 98; group B, 295; group C, 139; and group D, 103 persons. Overall, 12.3 and 18.4% of the population were seronegative for measles and rubella antibodies. This rate varied greatly between the 4 groups: group A, 0/0–2%; group B,15.2–25.0%; group C,11.5–17.2%; and groupD,14.6–18.4%. After revaccination, 92 and 94.9% of seronegative individuals showed IgG response to measles and rubella vaccines, respectively.

**Conclusion:**

Despite the high coverage rate of M-R containing vaccines, a significant number of vaccinated subjects were seronegative for measles and rubella, possibly because of secondary vaccine failure; this may negatively affect measles-rubella elimination targets in the country. If these findings are confirmed in similar future studies, a more robust regional/national supplementary immunization activity will be considered.

## Background

Measles, a highly contagious viral disease, is recognized as a major public health concern worldwide [1]. In 2018, there were more than 140/000 measles-related death globally, mostly among children under the age of 5 years [2]. Age of vaccination is one of the key host-related determinants of vaccine efficacy (VE). Results of previous studies have revealed that measles vaccination at the age < 12 months is associated with the reduced immune response rate due to interference of maternal antibodies and immaturity of the immune system [3–5]. To prevent the measles virus transmission in a community, a population immunity rate of > 93–95% with > 95% vaccination coverage (with two-doses of vaccine) is required in all districts of the country [1, 6]. In recent years, the number of measles cases and its outbreaks have started to increase, even in countries with history of disease elimination; also, some of these cases have been reported in fully vaccinated individuals [7–16]. From January to the end of July of 2019, a total of 364, 808 measles cases were reported to World Health Organization (WHO) by 182 countries, which has surpassed the number of reported cases (129. 239) within the same period in 2018 [7].

Rubella is a mild viral infection that affects unvaccinated children and adults. If a nonimmune woman contracts rubella during pregnancy, especially in the first trimester, serious consequences including miscarriage, fetal death, stillbirth and congenital rubella syndrome (CRS) may occur. Nonetheless, CRS is a vaccine-preventable congenital anomaly [17].

During the pre-vaccine era in Iran, measles and rubella were endemic and nearly 150,000–500,000 cases of measles with a high mortality rate were reported annually [18, 19]. However, more than three-fourth of adolescents and childbearing age women naturally acquired anti-rubella immunity [20, 21], and the incidence of CRS was estimated to be two per 10,000 live birth [22]. Fallowing the establishment of the WHO Expanded Program of Immunization (EPI) in Iran in 1984, the incidence of measles cases reduced markedly. However, in response to the increased number of measles cases particularly in older age groups, a nationwide measles- rubella (MR) immunization campaign targeting 5–25 year-old individuals was stablished in December 2003 to prevent CRS [23].

After the mass MR immunization program, many seroprevalence study results revealed that nearly 63.2–92% and 87–99% of vaccinees acquired seroprotection against measles and rubella respectively [24–29].

Since March 2004, the Mmv has been replaced with two-doses of measles-mumps-rubella (MMR) vaccine, scheduled after the age of 12 months, with more than 95% coverage rate [23, 30]. This shift in the program led to the accumulation of a birth cohort born between November 1998 to March 2004, who were vaccinated with two- doses of mMV at the ages of 9 and 15 months respectively. However, to provide protection against mumps and rubella, this particular group was revaccinated with one dose of MMR vaccine at the time of school entrance (2 to 5 years after the last dose of mMV).

In 2019, WHO Region Verification Committee of the Eastern Mediterranean declared the elimination of the measles and rubella in Iran [31]. However, in recent years, small outbreaks of measles have been reported in some parts of the country [12–16] Table 1. The measles virus causing these outbreaks was reported to originate from neighbor countries [16, 32].

**Table 1:** Measles outbreaks reported in Iran from 2006 to 2016. 2–14 years after the MR campaign and the onset of two-doses MMR immunization program

Author/ reported province	Years of outbreaks	No of cases	Involved age groups	vaccination status
Nejati J, et al./ Sistan- Baluchestan [12]	2006–11	456 cases	All age groups: 48% in < 5 yrs. 34% in 5–9 yrs	One dose: 11.2% 2-dose: 27.8%, not vaccinated: 55.1%
Izadi s southeast of Iran [13]	2009–10	126 cases (2 main outbreaks)	All age groups 42% ≥ 7 yrs. 6.3% > 20 yrs	2-dose vaccine efficacy =74.2%
Moghaddam [14] Fars	2012	7 cases	11mo - 35 yr	2-dose: 3case, Non-vaccinated: 2 cases
Karami M [15] National level	2012–2014	2012:232 cases 2014: 142 cases	All age groups	22.7%: < 12 mo 19.3%: vaccinated 36.5%: non-vaccinated
Piri N [ 16] National level	2014–2016	759 cases	31.1%: < 1 yr 13.2%: 1–4 y	20.4% < 12 mo 23.3%: vaccinated

There is little Information available regarding level of measles and rubella protection among children, adolescents and adults vaccinated with different schedules. Therefore, this study was designed to investigate the prevalence rates of measles and rubella immunity among various age groups that were vaccinated with different programs, and also to determine the relative role of secondary vaccine failure as a possible cause of serosusceptibility to viruses.

## Methods and participants

This descriptive-analytical cross-sectional study was conducted in the East of Mazandaran province, North of Iran, from 1-November 2017 to 30-June 2018. This region consists of three main districts with an approximate population of 460,000. Depending on the population density of each district, some primary health care centers (PHC) have been established. All basic health requirements including prenatal care and vaccination of children in the affiliated families are met in these centers. Also, each family has a specific file where all health related events of that family are recorded.

To select the eligible individuals, the family files available in each PHC were thoroughly reviewed. Healthy subjects who had born between years 1984 to 2011 with a documented history of measles vaccination were chosen. Based on the population density of each PHC, the subjects required for this study purpose were selected among assigned eligible persons with a simple random sampling method (one from two/ three eligible subjects). Since the majority of selected cases were students (primary and high school students), their vaccination status was rechecked from a copy of their booklet record which was present in their school file; the history of MR vaccination in some adults was based on their self- report. Individuals with acute diseases, history of recent febrile exanthematous illnesses, chronic and metabolic illnesses, malignancies, immunodeficiency, recipients of blood/ blood products within last year, recipients of additional dose of measles containing vaccine after the recommended schedule (except those who received MR vaccine during nationwide measles-rubella campaign immunization and recipients of MMR vaccine at school entrance among birth cohort 1998–2003) and pregnant women were all excluded from the study.

Depending on age and vaccination status, the subjects were categorized as follows; Group A: the subjects were born 1984 to October 1998, (age range: 20–33 years). Most of them were vaccinated with 1–2 doses of measles vaccine at the ages of 9 and 15 months and were also reimmunized with MR vaccine during the national MR Immunization campaign. Overall, they were vaccinated with three doses of measles and one dose of rubella vaccine. Group B: the subjects were born from November 1998 to March 2004 (age range: 15–19 years). This birth cohort was vaccinated with two doses of mMV at the ages of 9 and 15 months, respectively. They were also reimmunized with additional dose of MMR vaccine at the age of school entrance (age of 6 years); therefore, they received three doses for measles and one dose for rubella and mumps vaccine.

Group C: The subjects were vaccinated from March 2004 to March 2007 (Age range: 11–14 years). They were vaccinated with two doses of MMR vaccine at the ages of 12–15 months and 6 years, respectively. Group D: the subjects were vaccinated from April 2008 to the end of year 2011 (Age range 7–10 years) with two-doses of MMR at the ages of 12 and 18 months. The group characteristics are presented in Table 2.

**Table 2:** Demographic characteristics of studied population based on age, vaccination status, and time elapsed since receiving the last doe of vaccine, in the East of Mazandaran, North of Iran

Studied groups	A	B^d^	C^e^	D^f^	Total
Variable					
No of subjects	98	295	139	103	635
Age Range yr (Date of birth)	20–33,1984–1998	15–19,1999–2003	11–14,2004–2007	7–10,2008–2011	7–33,1984–2011
Female/male ratio	59/39	139/156	75/64	49/54	322/313
Year and Time elapsed since the last dose of vaccination	2003 /15 yr	2004–7/ 13–15 yr	2004–7/ 10–13 yr	2008–11/ 7–10 yr	–
history of MV_1_ and MV_2_^a^	+	+	–	–	-
History of MR^b^	+	–	–	–	–
History of MMR^c^	No	One dose	2-doses	2-doses	–

^a^: MV_1, 2_ measles vaccine first dose, second dose.

^b^: MR: measles-rubella vaccine.

^c^: MMR measles-mumps-rubella vaccine.

^d^: GB: these subjects, in addition to receiving two doses of MV at the ages 9 and 15 months, were immunized with one dose of MMR vaccine at time of school entrance.

^e^: This group was immunized with two–doses of MMR vaccine at the ages 12–15 months and 6 years.

^f^: This group was immunized with two–doses of MMR vaccine at the ages of 12 and 18 months.

We adhered to standard protocols and ethical guidelines in this study. The study was approved by the Ethic committee of Mazandaran and Tehran Universities of Medical Sciences: IR. MAZUMS. Rec.1396.3074 and Tehran IR. TUMS.IKHC. Rec.1399.075, respectively.

After obtaining informed written consent from guardians or individuals, 5 ml of venous blood was collected from all participants. The sera were stored at − 20 °C to measure anti-measles and anti-rubella lgG antibodies qualitatively at the university laboratory by ELISA method using Vircell Microbiologic ELISA measles and rubella lgG/lgM kits (vircell, S. L. parquet Technologico dela salud. Avecina 8. 18,016 Granada. Spain). Antibodies against measles and rubella were measured based on manufacturer’s instructions. The results were interpreted as antibody indices. The antibody indices were calculated with positive and negative controls (OD > 0.9 and OD < 0.5) at a control cut-off range of > 0.55 to < 1.5. Antibody Indices were calculated as: (Sample OD/cut-off serum mean OD) × 10. Samples with antibody indices above 11 were considered as positive (having specific measles IgG/IgM per procedure), while those with antibody indices less than 9 were considered as negative. Subjects with indices between 9 and 11 were rechecked and if reported > 11, were considered as positive and if reported < 11, were considered as negative. Similar categorization was applied for rubella. Nevertheless, rubella ELISA IgG has been standardized against WHO first international standard for antirubella IgG with a cut-off set at 10 UI/mL. Based on the manufacture’s reports, compared with other commercial ELISA specific kits, the sensitivity and specificity of kit for measles IgG were 92–100% and 97–99% and for measles IgM were 87–100% and 92–100% respectively. These rates for rubella IgG were 85–99% and 85–99%, and for rubella IgM were 87–100% and 93–100%, respectively. The mean concentration of antibodies (MCAs) for both viruses in seropositive subjects were calculated in each group. The proportion of seropositive individuals was calculated for all groups and within each group. Seronegative persons were revaccinated with one dose of MMR vaccine. Four to six weeks after boosting, the subjects’ sera were tested for measles and rubella specific lgM and lgG. Subjects with both lgM and lgG seroconversion were considered as cases of primary vaccine failure [possibly never develop immune response to the initial vaccination: (PVF)] whereas subjects with only lgG seroconversion were considered as secondary vaccine failure [loss of acquired specific antibody with time (SVF)]. MCAs of seropositive individuals were calculated after boosting, and were compared with the previous level. The collected data were analyzed using SPSS version 16.0. Descriptive statistics were measured as percentile for seropositivity and response rate to revaccination. The chi-square and student t-test were used to determine differences between variables as appropriate. The level of statistical significance was set at *P* < 0.05 .

## Results

A total of 635 individuals participated in this study. The demographic characteristics and vaccination status of the participants are presented in Table 2.

Of 635 studied subjects, 78 (12.3%) and 117(18.4%) were serologically susceptible to measles and rubella respectively. The prevalence rates of measles and rubella susceptibility in different age groups varied significantly, as shown in Table 3. The highest rates of susceptibility to measles and rubella were seen in the group B (15.3 and 25.0% respectively), followed by the group D (14.6 and 18.4%, respectively). The highest seroprotection rates (98 and 100%) were observed in oldest subjects, who were MR revaccinated. However, comparison of the prevalence rates of measles and rubella seroimmunity between the groups showed significant differences between group A and other groups: (*P* = 0.000). Also, there were significant differences in the measles MCA between group A and group B (*P* = 0.006) and between group A and group C individuals (*P* = 0.001). Similarly, significant differences were observed in rubella MCA between group A and group D (*P* = 0.013) and between group C and group D (*P* = 0.031).

**Table 3:** Measles and rubella immunity status among different groups vaccinated with various vaccination schedules and their response to revaccination, East of Mazandaran province, North of Iran

Groups (n) birth date	GA: *n* = 98,1984–1998	GB: *n* = 295,1999–2003	GC^a^: *n* = 139,2004–2007	GD: n = 103,2008–2011	Total *n* = 635,1984–2011
vaccination status:	A	B	C	D	different schedules
mMV:	+	+	–	–	
MR:	+	–	–	–	
MMR:	–	+ one dose	+ 2 doses	+ 2 doses	
Measles:	96 (98%)	250 (84.7%)	123 (88.5%)	88 (85.4%)	557 (87.7%)
Seroimmune n (%) MCA ± SD Response rate to revaccination respond/total(%)	25.15 ± 6.47	17.63 ± 7.21	19.34 ± 7.02	17.17 ± 7.42	19.19 ± 7.59
	–	26/28(92.8%)	11/12(91.6%)	9/10(90%)	46/50(92%)
Rubella:	98(100%)	221(74.9%)	115(82.9%)	84(81.55%)	518(81.57%)
Seroimmune n (%)MCA ± SD	25.71 ± 2.29	23.07 ± 15.56	24.67 ± 12.51	20.09 ± 9.83	23.36 ± 13.14
Response rate to revaccination respond/ total(%)	–	34/36(94.4%)	15/16(93.7%)	7/7(100%)	56/59(94.3%)

*mMV* Monovalent measles vaccine, *MMR* Measles-mumps-rubella vaccine, *MR* Measles rubella vaccine, *yr* Year, *Mo* Month, *MCA* Mean concentrations of antibody.

^a^: these 2 groups (C&D) that were vaccinated with 2 doses of MMR vaccine after the age of 12 months: 16 + 15 = 31 (12.8%) and 24 + 19 = 43(17.7%) were susceptible to measles and rubella respectively.

After revaccination of 171 susceptible cases (measles 78 and rubella 117), only 71 subjects (measles 50 and rubella 59) agreed to have blood sample collection for reevaluation. As shown in Tables 3, 92 and 94.9% of revaccinated seronegative persons responded well to MMR vaccine boosting and became IgG seroconverted against measles and rubella, respectively. None of the boosted subjects showed evidence of anti-measles or anti-rubella IgM response; therefore, seronegativity was possibly caused by loss of acquired immunity over time and SVF. The MCA levels of seroimmune individuals for both measles and rubella were not statistically significant after revaccination as compared to MCA levels in the primary evaluation: for measles 18.35 vs 20.06 *P* = 0.149, and for rubella; 22.63 vs 22.03, *P* = 0.603.

## Discussion

This study showed that more than 12 and 18% of the studied individuals were serologically susceptible to measles and rubella, respectively. The highest rates of susceptibility to measles and rubella (15.2 and 25% respectively) were observed among subjects in group B (15–19 year- old) who were born within 5 years before national MR immunization program and were vaccinated initially with two doses of mMV at the ages 9 and 15 months, respectively. Two to five years later, they also received an additional dose of MMR vaccine right before entrance to school (age of 6 years). Moreover, the present results showed that 12.8 and 17.7% of subjects who were vaccinated with two doses of MMR vaccine after the age of 12 months (group C and group D), were serologically susceptible to measles and rubella, respectively. In this study the lowest rate of serosuseptibility to measles and rubella was detected among 20–33- year- old adults who were MR revaccinated. Isolated lgG immunologic response to MMR revaccination in boosted susceptible individuals indicates that the main possible cause of susceptibility to measles and rubella in our vaccinated population was waning of acquired seroprotection with time (SVF). Moreover, the results revealed that the levels of acquired MCA after revaccination of seroimmune subjects for both measles and rubella with MMR vaccine, did not improve specific immunity to these viruses.

The present results showed that 98 and 100% of subjects in group A (age 20–33 years) who were covered by the national program of MR immunization were serologically immune to measles and rubella, respectively. The long- term high- rate of protection could be attributed to MR vaccination or natural boosting in recent years. Years before the MR campaign, the measles seroprevalence rates in the Iranian population were much lower [33–37] (40.7% [33] ^-^ to 91.6% [37]) than levels reported in studies performed years after revaccination in different age groups (63.2 [29] % -to 91.7 [28] % for measles and 87.4 [28] %-to 99 [24] % for rubella) [24–29]. The relevant data are presented in Table 4. In a recent nationwide study conducted 13–14 years after the national MR campaign on girls above 15 years, the seroprotection rates to measles and rubella were investigated. Nearly 1573 sera were included from ten different provinces. The seroimmunity rates to measles and rubella were 80.7% (range 73.1%- to 89.8%) and 90.6% (range; 81.2–95%) respectively [38], however, these rates varied greatly between provinces. The relatively high rate of seroprotection observed in our study as well as the mentioned studies, which were conducted years after national campaign could be attributed to the positive impact of MR revaccination and/or natural boosting in the immunized population.

**Table 4:** Measles and Rubella seroprevalence rates demonstrated in different studies before and after MR campaign in Iran

Author/province	Relation to 2003 MR campaign	Years of study	No of Subjects (Age-groups)	Tested method	Prevalence Rate	
					Measles	Rubella
Emami-Naeini, Shiraz [33]	Before 3-yr	2000	241 (19–25 yr)	ELISA	40.7%	
Yekta, Uremia [34]	Before (1- yr)	2002	835 (5–25 yr)	ELISA	54.7%	–
Saffar, Sari [35]	Before (1- yr)	2002	590(15–25 yr)	ELISA	55.4%	–
Zamani, Tehran [36]	Before (2- yr)	2001	1665 (6-11 yr)	ELISA	72%	–
Salimi, Tabriz [37]	Before (1- yr)	2002	225 (5–25 yr)	HI test	91.6%	–
Pourabbas, Shiraz [24]	After (1- yr)	2004–5	909(6-26 yr)	ELISA	80.6–87.5%	91.0–99%
Yekta, Uremia [25]	After (1- yr)	2004	624 (6-25 yr)	ELISA	72.3%	–
Honarvar, Shiraz [26]	After (7- yr)	2010–11	175 (16-24 yr)	ELISA	81.7%	96%
Keshavarz, Tehran [27]	After (10- yr)	2014	53 (19–26 yr)	ELISA	79.2%	96.2%
Izadi, Bluchestan [28]	After (12–13- yr)	2015	1056 (16–20 yr)	ELISA	91.7%	87.4%
Karami, Hamadan [29]	After (13- yr)	2016	272 (1–40 yr)	ELISA	63.2%	–

*Yr* Year, *mo* Months.

In this study, the highest rate of measles and rubella susceptibility was observed in subjects of group B (age range: 15–19 years), who were vaccinated not only with two- doses of mMV at the ages of 9 and 15 months, respectively, but also received an additional dose of MMR vaccine upon school entrance (three doses of measles and one dose of rubella vaccine). This seronegativity to MR viruses in this age group detected nearly 13–15 years after the last dose of MMR vaccine, is unusual and raises some concern. Since, there is no information about immune responses to the primary measles immunization in this age group, the actual reasons for this rate of susceptibility and vaccine failure is unclear. However, waning of acquired seroimmunity over time may be influential, as the majority of boosted susceptible subjects in this group only showed IgG response to MMR revaccination.

The quality and durability of measles vaccine-induced immunity are dependent on a number of factors including the host and the vaccine status. The most important and well-studied host-related determinant is the age when the first dose of vaccine is administered [3–5, 39]. Studies on the immunogenicity and VE of MV, administered before the age of 12 months, showed lower rates when compared to older ages [3–5, 39]. In this regard, a prospective randomized trial by Redd et al. [3], investigated the immunogenicity of measles component of MMR vaccine administered at the ages 9,12, and 15–18 months. They found a 98% seroconversion rate among 15 month-old vaccinees as compared to 95% in those vaccinated at the age of 12 months and 81% in those vaccinated at the age of 9 months [3]. Moreover, a study by perez et al. [4] revealed that measles vaccination at the age of < 12 months was associated with a higher risk of PVF. This negative effect persisted even after vaccination with the second dose [4]. These results were also supported by other recent systematic reviews and meta-analysis [5, 39]. Moreover, to determine the effects of age on the immunogenicity of measles vaccine, a systematic review was recently performed. This study showed that earlier age of vaccination decreases measles immunogenicity and protection after the first dose, and could still have influence on VE after two-doses of measles vaccine [39]. However, in another review by Uzicanin et al. [5], estimating the measles VE, they found that two-doses of measles vaccine provided excellent protection; Nevertheless, in three out of eight studies of this review article, VE was estimated at < 90% compared with no vaccination [5].

On the other hand, some evidence suggests that antibody concentrations decline to low or undetectable levels over time [40–44]. In this regard a study was conducted on different age groups of Iranian children who were vaccinated against measles at the ages of 9 and 15 months. The seroimmunity rates were 52.9 and 89.2% at five and three months after administering the first and second doses of MV, respectively. These rates decreased to 68% by the age 6 years and to 40.5% by the age ten years. However, the seroimmunity rates increased to 96.8% at 9 months after boosting with one dose of MV at the age of 14 years [40]. Moreover, in a longitudinal study by Kremer et al. [41], on the kinetic of measles and rubella antibodies, both antibodies waned over time, also, the rate of waning immunity was relatively faster for measles [41]. Considering the relatively high rates of measles and rubella susceptibility in group B of our study, this seronegativity could be attributed to both primary and secondary vaccine failure. To determine whether the seronegativity is related to PFV or SVF, there are two methods of assessment. These methods include IgG avidity test and IgM response to revaccination. In this study, we used the IgM method, and there was no positive response. These negative results are probably due to SVF. Possibility of delay in blood sampling for IgM detection and the low sensitivity of the assay may be also influential. Nevertheless, isolated IgG seroconversion in boosted seronegative subjects is most probably related to SVF.

The results of most studies from developed countries have shown that approximately 90–95% of children vaccinated at the age of ≥12 months produce sufficient specific antibodies against measles and rubella. The protection rates will increase up to 95–98% after the second dose vaccination and persist for decades [1, 3–6], although results of some studies have shown that the achieved seroprotection rate may decline over time or years after the initial immunization [42–44]. In this study, nearly 12.8 and 17.7% of 7–15-year-old subjects in group C and D (vaccinated with two doses of MMR vaccine administered after the age of 12 months) were serologically susceptible to measles and rubella, respectively, however, the exact cause of this lower unexpected rate is unknown. After revaccination, nearly all boosted serosusceptible subjects develop isolated IgG antibody response without IgM, and showed evidence of secondary immune response beside waning of acquired immunity over time. Furthermore, in the present study, waning of measles and rubella seroprotection rates after the primary vaccination occurred relatively earlier than expected [1, 5, 6, 39]. Loss of acquired immunity after vaccination, particularly within a shorter period than expected time, is of concern [45, 46]. Therefore, vaccine- related factors such as lack of adequate potency of vaccine because of using more thermo- labile strains, inadequate control of cold chain shipment, storage, use, and other probable factors may be responsible [45–47]. Our assumption regarding the inadequate potency of vaccine is based on the results of studies that were designed to investigate the immunogenicity of MMR vaccine currently used in Iran [48–53]. The majority of these studies showed lower rates of seroconversion following the first and/or the second doses of MMR vaccine after the age of 12 months (Table 5).

**Table 5:** Immunogenicity and seroconversion rates to measles and rubella components of MMR vaccine currently used in Iran

Author/province vaccine brand	Years of study	No of Subjects	Age	Lab method	Responses Rate MMR (%)			
					MMR1^first dose^		MMR2^2second dose^	
					M	R	M	R
Saffar, Mazandaran Razi-Iran [48]	2007	112	12.10 mo	ELISA	84.8%	53%	–	–
Saffar, Mazandaran Razi-Iran [49]	2011	249 228	18 m 6 yr	ELISA	74% 6- mo after MMR1 5- yr after MMR1 78.9%	75%6- mo afetr MMR1 66% 5- year after MMR1	94.4%	92.6%
							1- mo after MMR2	
							98.2	87%
							1- mo after MMR2	
Shamsizadeh, Ahwaz Karaj-Iran Razi Iran [50]	2010–2011	70 90	18 mo 6.5 yr ^a^	ELISA	6- mo after MMR1 42.9% -	6- mo after MMR190% -	- 45.6%	- 87.8%^a^
Tabatabaei, Razi-Iran [51]	2011–2012	240	13.27 mo [12–15]	ELISA	75.8%	73.8%	–	–
Zahrari, Baluchestan –Kerman-Hormozgan Razi-Iran [36]	2016	236	> 12 mo	ELISA	91.2%^b^		–	
Izadi, Baluchestan –Kerman-Hormozgan Razi-Iran [37]	2015	663	30–54 mo		–	–	94.6%^c^	–

^a^: One dose of MMR vaccine in addition to two doses of MV at the ages nine and 15 months.

^b^: Based on strict control of the vaccination administration technique and cold chain control by the researchers.

^c^: Two- doses of MMR at the ages of 12 and 18 months.

Reduced levels of measles- rubella antibodies in the post- vaccinated period, may result in the accumulation of potentially susceptible individuals to measles and/or rubella in the community. Several reports have described significant rates of SVF in population with a sustained high rates of vaccination coverage and long absence of measles virus transmission [40–44]. In this regard, a prospective multicenter study performed by Smetana et al [43], evaluated the measles lgG antibody concentration among vaccinated subjects ≥18 years. Of 1911 sera, 83.3% were seropositive. When different age groups were compared, the seroprevalence rate decreased overtime (18–29 years: 81.1% and 30–39 years: 61.5%). The results of a similar study in Korea also indicated a progressive decline in level and avidity of antibodies over time in 2- to 30-year-old vaccinated individuals [44]. Investigation of the measles outbreaks indicated the vaccine failure in 11–49% of measles cases in several large outbreaks [11, 54–57]. Also, in an epidemic, up to 14% [56] of cases had received at least two-doses of measles vaccine. These findings suggest SVF as the main cause of susceptibility which was mentioned earlier in group C and D of our study. Since the development of SVF was faster in these groups compare to other studies, further investigations are recommended to evaluate the immunogenicity and long-term protection of measles vaccine in the Iranian population.

The WHO Regional Verification Commission of the Eastern Mediterranean region for measles and rubella elimination declared elimination of measles and rubella in Iran [31]. In the present study among 7- to 33 year- old individuals, who were vaccinated at least with two-doses of measles vaccine with different schedules, nearly 87 and 81% were seroprotected to measles and rubella respectively. Considering a 95% coverage rate with two-doses of vaccine, immunity rates of 83 and 77.6% were estimated in the population respectively. This level of immunity is less than what is necessary to prevent measles and rubella virus transmission in the community and achieve disease elimination (93–95% and 88–90% for measles and rubella respectively) [1, 6]. Also, phylogenetic analysis of the isolated measles viruses in the outbreaks of Iran showed major similarity with the measles viruses detected in neighbor countries. In some of these countries measles is still endemic [16, 32], which can be an alarming sign for Iran.

Therefore, further long-term prospective studies are recommended to evaluate the immunogenicity of MMR vaccine and investigate the persistence of seroimmunity. If the present study results are confirmed in further studies, additional dose of MMR vaccine will be required as a national/regional supplementary immunization activity program for the age group of 10–25-year-old individuals to sustain measles-rubella elimination in Iran [38].

One of the limitations of this study is the lack of information about the seroimmunity status after the primary vaccination which can differentiate between PVF and SVF. Also, the method used for the assessment of IgM response to revaccination may not be sensitive enough. Another limitation of this study is that it was not designed as a population based study, it was carried out in East of Mazandaran province (north of Iran) with a modest number of participants which made the results less generalizable. Finally, there may be recall bias in group A regarding MR vaccination.

## Conclusions

Based on the findings, nearly 12.3 and 18.4% of fully vaccinated individuals, aged 7–33 years, were seronegative to measles and rubella, respectively. SVF was recognized as the main cause of seronegativity to measles and rubella. The level of seroprotection detected in this study was lower than what is required to achieve/maintain the elimination goals. To sustain measles and rubella elimination in Iran, further studies are recommended to assess the immunogenicity of the current MMR vaccine and strictly monitor the vaccine cold chain in all stages until use. Finally, periodic serosurveillance studies must be designed for different age groups in different provinces of Iran to detect gaps in the population immunity.

## Data Availability

Obtained for this study will be available from the corresponding author at a reasonable request.

## Abbreviations

mMV: Monovalent measles vaccine
MV: Measles vaccine
MMR: Measles mumps rubella
EPI: Expanded Program of Immunization
CRS: Congenital rubella syndrome
PVF: Primary vaccine failure
SVF: Secondary vaccine failure
WHO: World Health Organization
MAZUMS: Mazandaran University of Medical Sciences
TUMS: Tehran University of Medical Sciences
ELISA: Enzyme linked immunosorbent assay
IU/L: International unit/ liter
IgG: Imunoglobulin G
IgM: Immunoglobulin M
MCA: Mean concentration antibodies
PHC: Primary health care center
VE: Vaccine efficacy
